# Studies on sequential metabolic alterations in spontaneous mammary carcinogenesis.

**DOI:** 10.1038/bjc.1967.25

**Published:** 1967-03

**Authors:** N. A. Sheth, M. M. Wagle, S. V. Bhide, K. J. Ranadive


### STUDIES ON SEQUENTIAL METABOLIC ALTERATIONS IN SPONTANEOUS MAMMARY CARCINOGENESIS

NANDINI A. SHETH, MANJULA M. WAGLE*, SUMATI V. BHIDE AND KAMAL J. RANADIVE

*From the Biology Division, Indian Cancer Research Centre, Parel, Bombay, 12, India*

Received for publication October 6, 1966

VOLUMINOUS literature is available on biochemical differences between malignant and non-malignant tissue, yet, precise biochemical characterisation of carcinogenesis of tissue is still a challenge to the biochemists at large. In recent years a number of biochemical studies on induced carcinogenesis of liver have been reported (Weber, Banerjee and Morris, 1961). However, literature on spontaneous carcinogenesis of any other tissue (including mammary tissue) is very scanty. The importance of experimental work on mammary carcinogenesis in general, and of systematic biochemical studies of the events before development of breast cancer in particular, need not be emphasised. Extensive biological studies on sequential histological development in mammary tumorigenesis have been reported from this laboratory (Ranadive, 1959) and abroad (Severi, 1958). The present authors have been interested in mammary carcinogenesis to study metabolic changes associated with malignant transformation and correlation of significant biochemical changes concurrent with the formation of precancerous hyperplastic nodules in the breast tissue.

The present paper reports salient observations on metabolic alterations taking place in the breast tissue of mice from young adult stage of 4 months (when the mammary tissue shows normal histology) till the development of tumour in two strains of mice susceptible to breast cancer. For this purpose metabolic parameters of growth such as levels of ribo- and deoxyribonucleic acids, activities of ribonuclease (RNAase), adenosine-triphosphatase (ATPase) and acid and alkaline phosphatases were measured. Mammary glands of 4, 6, 8 and 10 months old virgin mice of two susceptible strains namely C3H (Jax) and I.C.R.C. were used for the experimental purpose. The latter strain, i.e. I.C.R.C. strain, is a newly developed inbred line of albino mouse and is highly susceptible to breast cancer (Ranadive and Kanekar, 1963). Virgin mice of corresponding age groups of C57 (Black) strain resistant to breast cancer were used for comparison.

### MATERIALS AND METHODS

Virgin mice of two strains C3H (Jax) and I.C.R.C. both highly susceptible to spontaneous breast cancer and a resistant strain C57 (Black) were divided into the following age groups : (1) 4 months old, (2) 6 months old, (3) 8 months old, (4) 10 months old and (5) the last group consisted of tumour-bearing mice of the two susceptible strains only.

* Present address : Biology Group, Atomic Energy Establishment Trombay, Richardson and Crudas Building, Byculla, Bombay, 8.

Animals were killed group-wise, and breast tissue was removed carefully. The tissue was homogenised in a Potter Elvehjem homogeniser in distilled water to make it to 10 per cent. In tumour bearing mice tumour tissue instead of breast tissue was used for estimations. The tumour bearing animals (most of them between the age of 10 and 12 months) were killed when the tumour was 2 × 2 cm. in size. Tumour tissue was also homogenised in distilled water to make it to 10 per cent.

The homogenate was used for the following estimations :

1. Estimation of ribonucleic acid by Ogur and Rosen's method (1950).
2. Deoxyribonucleic acid was estimated by indole method (Ceriotti, 1952).
3. Tyrosine was estimated colorimetrically by the method of Lowry *et al.* (1951).
4. Estimation of acid ribonuclease was done by treating the homogenate with

RNA solution (3 mg./ml.) in acetate buffer pH 5·2. The reaction was stopped by adding 10 per cent perchloric acid in alcohol at −10° C. and excess of RNA was precipitated. The activity was expressed as μg. of nucleotides liberated by the action of RNAase per μg. of tyrosine.

5. Activities of acid and alkaline phosphatases were measured using sodium-β-glycerophosphate as substrate at pH 5·2 and 9·2 respectively. Phosphorus was estimated by Weil-Malherbe and Green's method (1951). Activities were expressed as μg. of phosphorus per μg. of tyrosine.

6. Adenosine triphosphatase (ATPase) activity was measured by the method of Krishnan (1955). In this case also μg. of phosphorus liberated per μg. of tyrosine denoted the enzyme activity.

All the results presented in this paper are the mean values of a minimum of six experiments.

### OBSERVATIONS

Table I shows the contents of ribonucleic acid in mammary glands of virgin mice of three strains, namely C3H (Jax), C57 (Black) and I.C.R.C., at different

TABLE I.—*Content of Ribonucleic Acid in Breast and Tumour Tissues of C3H (Jax), C57 (Black) and I.C.R.C. Strains of Mice in Different Age Groups*

| Strain | Age groups | | | | |
|---|---|---|---|---|---|
| | 4 months | 6 months | 8 months | 10 months | Tumour bearing |
| C3H (Jax) . . | 0·132±0·02 (100%) | 0·147±0·005 (111%) | 0·26 ±0·03 (200%) | 0·208±0·02 (157%) | 0·22 ±0·044 (166%) |
| C57 (Black) . . | 0·16 ±0·03 (100%) | 0·19 ±0·05 (119%) | 0·15 ±0·03 (94%) | 0·109±0·025 (68%) | — — |
| I.C.R.C. . . . | 0·085±0·008 (100%) | 0·20 ±0·02 (235%) | 0·293±0·036 (344%) | 0·24 ±0·011 (282%) | 0·262±0·07 (308%) |

Content of ribonucleic acid is expressed in μg. of ribonucleic acid per μg. of tyrosine.
Values in brackets are expressed in percentages of the values of the breast at 4 months, which are taken arbitrarily as 100 per cent.

age periods. It is obvious that the patterns of RNA levels in the two susceptible strains are comparable. In both the strains RNA level is low at 4 months, and reaches its peak at 8 months. Further variations in the 10-month-old breast tissue and in tumour tissue are not statistically significant. It may be noted that

RNA content of the breast at precancerous stage at 8 months and that of the tumour is comparable (yet 60 per cent higher than that of normal breast tissue at 4 months). The C57 (Black) strain on the other hand does not have any significant difference in RNA content from 4 months to 10 months.

TABLE II.—*Content of Deoxyribonucleic Acid in Breast and Tumour Tissues of C3H (Jax), C57 (Black) and I.C.R.C. Strains of Mice in Different Age Groups*

| Strain | Age groups: 4 months | 6 months | 8 months | 10 months | Tumour bearing |
|---|---|---|---|---|---|
| C3H (Jax) | 0·25 ±0·028 (100%) | — | 0·37 ±0·05 (148%) | 0·41 ±0·02 (164%) | 0·43 ±0·04 (172%) |
| C57 (Black) | 0·49 ±0·05 (100%) | 0·43 ±0·14 (87·7%) | 0·406±0·05 (82·8%) | 0·505±0·04 (103%) | — |
| I.C.R.C. | 0·279±0·02 (100%) | 0·535±0·18 (191%) | 0·573±0·05 (205%) | 0·57 ±0·04 (204%) | 0·475±0·05 (170%) |

Content of deoxyribonucleic acid is expressed in μg. of deoxyribonucleic acid per μg. of tyrosine.
Values in brackets are expressed in percentages of values of the breast at 4 months which are taken arbitrarily as 100 per cent.

It may be noted from Table II that in both the susceptible strains DNA content is lowest at the age of 4 months. The tumour has a significantly higher DNA level than the normal breast tissue at 4 months. In C3H (Jax) strain DNA content increases steadily from the age of 4 months until the formation of tumour. However, in the I.C.R.C. strain the significant increase in DNA content (from 100 to 191 per cent) is noted at the age of 6 months ; thereafter there is no statistically significant variation in the latter age groups or in the tumour.

In the C57 (Black) strain there is no significant difference in DNA content of breast tissue from the age of 4 months to 10 months.

TABLE III.—*Activity of Ribonuclease of Breast and Tumour Tissues of C3H (Jax), C57 (Black) and I.C.R.C. Strains of Mice in Different Age Groups*

| Strain | Age groups: 4 months | 6 months | 8 months | 10 months | Tumour bearing |
|---|---|---|---|---|---|
| C3H (Jax) | 0·64 ±0·13 (100%) | 0·86 ±0·19 (134%) | 1· 43 ±0·08 (223%) | 0·35 ±0·09 (54%) | 0·17 ±0·05 (26%) |
| C57 (Black) | 0·53 ±0·15 (100%) | 0·65 ±0·04 (122%) | 0·71 ±0·25 (134%) | 0·64 ±0·04 (120%) | — |
| I.C.R.C. | 0·21 ±0·04 (100%) | 0·45 ±0·13 (214%) | 0·523±0·06 (249%) | 0·373±0·038 (177%) | 0·329±0·04 (156%) |

RNAase activity is expressed as μg. of nucleotides liberated per μg. of tyrosine.
Values in brackets are expressed in percentages of the values of the breast at 4 months, which are taken arbitrarily as 100 per cent.

Activities of RNAase of breast tissue at various age periods are shown in Table III. As in nucleic acids, the pattern of ribonuclease activity is similar in the two susceptible strains. It increases from the age of 4 months till the age of 8 months and then declines in 10 months and in the tumour bearing group. It may be noted that (i) in the C3H (Jax) strain the most significant rise (from 134 per cent to 223 per cent) in enzyme activity occurs at the age of 8 months and (ii) that the tumour RNAase activity is as low as 26 per cent of the normal breast tissue at 4 months age. In the I.C.R.C. strain the substantial increase from 100 per cent to 214 per cent is noted at the age of 6 months and the decrease in RNAase activity after 8 months is much less than that in the C3H (Jax) strain at corresponding age. Hence the tumour RNAase activity in the I.C.R.C. strain is statistically comparable with breast tissue at 4 months.

RNAase activity in the C57 (Black) strain does not significantly vary in all the age groups.

TABLE IV.—*Activity of Adenosine Triphosphatase of Breast and Tumour Tissues of C3H (Jax), C57 (Black) and I.C.R.C. Strains of Mice in Different Age Groups*

| | Age groups | | | | |
|---|---|---|---|---|---|
| Strain | 4 months | 6 months | 8 months | 10 months | Tumour bearing |
| C3H (Jax) . . | 0·07 ±0·02 (100%) | 0·056±0·02 (80%) | 0·083±0·02 (118%) | 0·16 ±0·03 (228%) | 0·14 ±0·025 (200%) |
| C57 (Black) . . | 0·077±0·01 (100%) | 0·08 ±0·01 (104%) | 0·11 ±0·02 (143%) | 0·077±0·02 (100%) | — — |
| I.C.R.C. . . . | 0·047±0·015 (100%) | 0·17 ±0·05 (361%) | 0·19 ±0·01 (404%) | 0·20 ±0·01 (425%) | 0·14 ±0·05 (297%) |

Activity of ATPase is expressed as µg. of phosphorus liberated per µg. of tyrosine.
Values in brackets are expressed in percentages of the vaues of the breast at 4 months, which are taken arbitrarily as 100 per cent.

Table IV shows the activities of ATPase in the three strains under study. ATPase activity in the C3H (Jax) strain remains comparable in 4, 6 and 8 months old groups. A significant increase is noted only at the age of 10 months and then remains comparable in the tumour. The tumour has 100 per cent more enzyme activity than the breast at 4 months age. In the I.C.R.C. strain, enzyme activity is lowest in 4 months old breast tissue. It rises significantly at the age of 6 months (from 100 per cent to 361 per cent) and then remains comparable in the later age groups as well as in tumour itself. The tumour has 193 per cent higher enzyme activity than normal breast tissue at 4 months.

In the C57 (Black) strain there is no significant change in ATPase activity in different age groups.

TABLE V.—*Activity of Acid Phosphatase of Breast and TumourTissues of C3H (Jax), C57 (Black) and I.C.R.C. Strains of Mice in Different Age Groups*

| | Age groups | | | | |
|---|---|---|---|---|---|
| Strain | 4 months | 6 months | 8 months | 10 months | Tumour bearing |
| C3H (Jax) . . | 0·355±0·04 (100%) | 0·37 ±0·045 (104%) | 0·421±0·065 (118%) | 0·445±0·85 (125%) | 0·476±0·09 (134%) |
| C57 (Black) . . | 0·347±0·046 (100%) | 0·37 ±0·01 (106%) | 0·42 ±0·06 (121%) | 0·29 ±0·063 (83%) | — — |
| I.C.R.C. . . . | 0·36 ±0·073 (100%) | 0·44 ±0·02 (122%) | 0·37 ±0·08 (102%) | 0·366±0·045 (100%) | 0·234±0·05 (65%) |

Acid phosphatase activity is expressed as µg. of phosphous liberated per µg. of tyrosine.
Values in brackets are expressed in percentages of the values of the breast at 4 months, which are taken arbitrarily as 100 per cent.

TABLE VI.—*Activity of Alkaline Phosphatase of Breast and Tumour Tissues of C3H (Jax), C57 (Black) and I.C.R.C. Strains of Mice in Different Age Groups*

| Strain | Age groups | | | | |
|---|---|---|---|---|---|
| | 4 months | 6 months | 8 months | 10 months | Tumour bearing |
| C3H (Jax) | 1·007±0·06 (100%) | 0·933±0·11 (92%) | 0·84 ±0·03 (83%) | 1·03 ±0·12 (105%) | 1·545±0·25 (153%) |
| C57 (Black) | 0·822±0·13 (100%) | 1·02 ±0·2 (124%) | 1·21 ±0·04 (148%) | 0·74 ±0·12 (90%) | — |
| I.C.R.C. | 1·06 ±0·13 (100%) | 1·08 ±0·1 (101%) | 0·997±0·07 (94%) | 0·91 ±0·09 (86%) | 0·876±0·14 (82%) |

Alkaline phosphatase activity is expressed as $\mu$g. of phosphorus liberated per $\mu$g. of tyrosine. Values in brackets are expressed in percentages of the values of the breast at 4 months which are taken arbitrarily as 100 per cent.

Acid phosphatase activity of all the three experimental strains is shown in Table V. In all the three strains, acid phosphatase activity does not change significantly in all the age groups.

In Table VI are shown the activities of alkaline phosphatase. In all the three strains it may be noted that the breast tissue of all age groups has very high alkaline phosphatase activity as compared with the corresponding acid phosphatase activity. Further it may be noted that alkaline phosphatase activity remains unaltered in all the age groups.

### DISCUSSION

Important metabolites which are studied in the present experiment may be grouped as follows :

Alkaline phosphatase—marker enzyme of the breast tissue and the breast tumour.

Ribo- and deoxyribonucleic acids—directly associated with growth and proliferation of the cells.

Ribonuclease and adenosine triphosphatase—associated with growth.

With reference to phosphatases it may be stated that the normal adult breast tissue possesses high alkaline and low acid phosphatase activity. Both the enzyme activities do not change appreciably in the higher age groups and in the tumour itself. The role of alkaline phosphatase in the mammary gland development and function is not yet clearly understood. Folly (1949) has postulated that it may play an important role in lactose biosynthesis. It is thus significant that alkaline phosphatase remains unaffected during malignant transformation of the breast and in the breast tumour itself. A similar case where the tissue specific enzymes are maintained during carcinogenesis of the tissue is that of slow growing hepatoma. Weber, Banerjee and Morris (1961) have shown that Morris hepatoma 5123 possesses glycolytic and gluconeogenic enzymes in the range of normal liver activity. In fact we have shown previously that mammary carcinomas in different strains of mice have higher alkaline than acid phosphatase activity, and alkaline phosphatase activity may be treated as a marker enzyme of the mammary tumours (Sheth *et al.*, 1966).

The salient points pertaining to other metabolites may be enumerated as follows : Contents of both the nucleic acids and activity of ATPase are lowest in the breast tissue of 4 months old virgin mice of both the susceptible strains, these then increase until the age of 8 months. Further it may be noted that the substantial increase in the graded rise of the entities occurs at 6 months in the I.C.R.C. strain and at 8 months in the C3H (Jax) strain. Thereafter they remain comparable in the later age groups and tumour bearing group. RNA, DNA and ATPase levels of the tumour are significantly higher than the normal breast tissue at the age of 4 months. This observation is amply supported by a number of investigators and need not be emphasised here (Greenstein, 1954; Aisenberg, 1961). With reference to RNAase it may be stated that in both the susceptible strains there is a considerable rise in enzyme activity from the age of 4 months to the age of 8 months. It then decreases in the tumour. Low RNAase activity in tumour has been reported by several workers (Roth, 1963). The increase in RNAase activity in early carcinogenesis of the hepatic tissue is reported by Cantero, Daoust and De Lamirande (1950). In view of recent emphasis on the role of lysosomal enzymes in carcinogenesis of tissue, the increase in RNAase activity is interesting. It has been reported that a number of lysosomal enzymes increase significantly in the early stages of liver injury (Slater, Greenbaum and Wang, 1963). It is possible that these lytic enzymes may play a role in the transformation of normal to malignant tissue. Further it is worth noting that the most substantial increase in the enzyme activity occurs at the age of 6 months in the I.C.R.C. strain (from 100 to 214 per cent) and at the age of 8 months in the C3H (Jax) strain (from 134 to 223 per cent). The increase in enzyme activity does not seem to be associated with ageing but with malignant transformation of the breast tissue because the virgin mice of C57 (Black) strain do not show any significant variation in enzyme activity in different age groups.

To appreciate the implications of these metabolic changes one has to consider the biological picture of the breast tissue at various age periods. At 4 months the mammary glands of all the three strains show a normal histological picture. In the majority of I.C.R.C. mice precancerous hyperplastic nodules appear around the age of 6 months (Ranadive and Kanekar, 1963), which is much earlier than in the C3H (Jax) strain. In the C3H (Jax) strain well defined precancerous nodules are developed around the age of 8 months (Ranadive, 1945). In the light of these biological observations, the substantial increases in RNA, DNA, ATPase and RNAase at the age of 6 months in the I.C.R.C. strain and at the age of 8 months in C3H (Jax) strain seems significant. Further it may be noted that following the appearance of these nodules, there is no significant change in the contents of these entities (except RNAase) in the later age groups, or in the tumour itself. In the present experiment all the metabolites are expressed per $\mu$g. of protein. Increase in cellular mass in tumour formation is accompanied by the increase in different entities, as well as in protein content, therefore an increase in absolute quantities may not be apparent in the later age groups or in the tumour itself. Hence, the metabolic changes which are concurrent with the formation of hyperplastic precancerous nodules stand out significantly. It is quite conceivable that the increase in these metabolites occurs at the time of specific inherent changes in the cell mechanism. These metabolic changes are probably controlled by alteration at genetic level in the normal mammary cells. Such cell transformation is then followed by the burst of proliferative activity leading towards the tumour formation. Of course it is essential to investigate further specific parameters or markers of mammary tissue, showing significant alterations at the precancerous nodular stage. It is reported that certain enzymes such as xanthine oxidase are rich in normal mammary tissue but are very low in the tumour (Bergel *et al.*, 1957). It would be interesting to see if this decrease in the enzyme activity occurs at the early transformatory stage or in the tumour itself. Studies on xanthine oxidase and other catabolic enzymes are in progress and will be reported later.

### SUMMARY

Breast tissue of 4, 6, 8 and 10 months old virgin mice of two strains, namely I.C.R.C. and C3H (Jax), both susceptible to breast cancer and of C57 (Black) resistant to breast cancer was studied. Content of ribo- and deoxyribonucleic acids and activities of acid and alkaline phosphatases, adenosine triphosphatase and ribonuclease were measured. It was observed that concurrent with the formation of hyperplastic nodules in the breast tissue of two susceptible strains around the age of 6 months in I.C.R.C. and at the age of 8 months in C3H (Jax), the levels of ribo- and deoxyribonucleic acids and ATPase activity increase significantly. Following the formation of hyperplastic nodules there is no significant change in the levels of these metabolites, in the breast tissue of the later age-groups or in the tumour itself. RNAase activity increases up to the formation of hyperplastic nodules and then decreases in the breast tissue of the later age-groups and in the tumour itself. In the resistant strain various metabolites of the breast tissue, do not change significantly at different age periods. Experimental observations are discussed in the light of relevant literature.

### REFERENCES

AISENBERG, A. C.—(1961) 'The Glycolysis and Respiration of Tumours'. New York and London (Academic Press), p. 149.

BERGEL, F., BRAY, R. C., HADDOW, A. AND LEWIN, T.—(1957) *Ciba Fdn Symp.* on The Chemistry and Biology of Purines. Edited by G. E. W. Wolstenholme and Cecilia M. O'Connor. London (Churchill), p. 256.

CANTERO, A., DAUST, R. AND DE LAMIRANDE, G.—(1950) *Science, N.Y.*, **112**, 221.

CERIOTTI, G.—(1952) *J. biol. Chem.*, **198**, 297.

FOLLY, S. J.—(1949) *Biol. Rev.*, **24**, 316.

GREENSTEIN, J. P.—(1954) 'Biochemistry of Cancer'. New York (Academic Press Inc.), p. 388.

KRISHNAN, P. S.—(1955) *Meth. Enzym.*, **2**, 591.

LOWRY, O. H., ROSENBOURGH, N. J., FARR, A. L. AND RANDALL, R. J.—(1951) *J. biol. Chem.*, **193**, 265.

OGUR, M. AND ROSEN, G.—(1950) *Archs Biochem.*, **25**, 262.

RANADIVE, K. J.—(1945) *Proc. Indian Acad. Sci.*, **22**, 18.—(1959) *Indian J. med. Res.*, **47**, 585.

RANADIVE, K. J. AND KANEKAR, S. A.—(1963) *Indian J. med. Res.*, **51**, 1005.

ROTH, J. S.—(1963) *Cancer Res.*, **23**, 657.

SEVERI, L.—(1958) *Int. Symp. on Mammary Cancer*, Perugia, 1958.

SHETH, N. A., WAGLE, M. M., BHIDE, S. V. AND RANADIVE, K. J.—(1966) *Br. J. Cancer*, **20**, 168.

SLATER, T. F., GREENBAUM, A. L. AND WANG, D. Y.—(1963) *Ciba Fdn Symp.* 'Lysosomes'. Edited by A. V. S. de Reuck and Margaret P. Cameron. London (Churchill).

WEBER, G., BANERJEE, G. AND MORRIS, H. P.—(1961) *Cancer Res.*, **21**, 933.

WEBER, G.—(1962) *Adv. Cancer Res.*, **6**, 403.

WEIL-MALHARBE, H. AND GREEN, R. H.—(1951) *Biochem. J.*, **49**, 286.


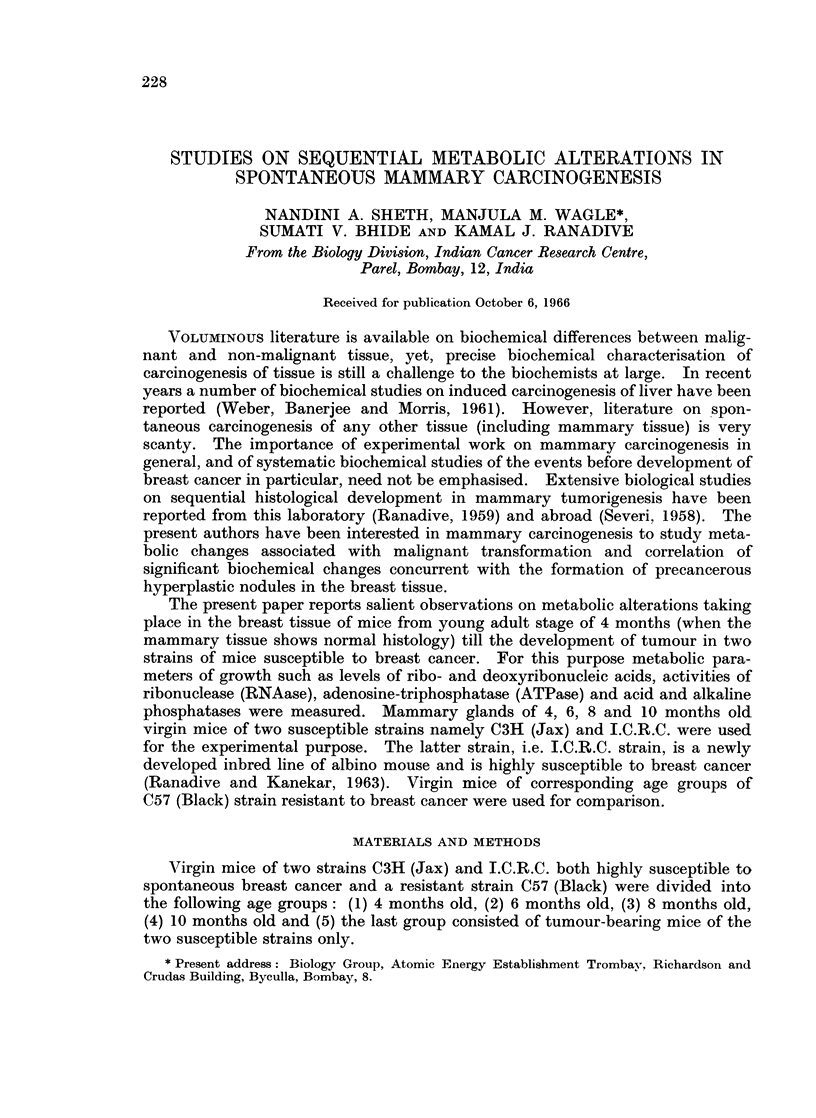

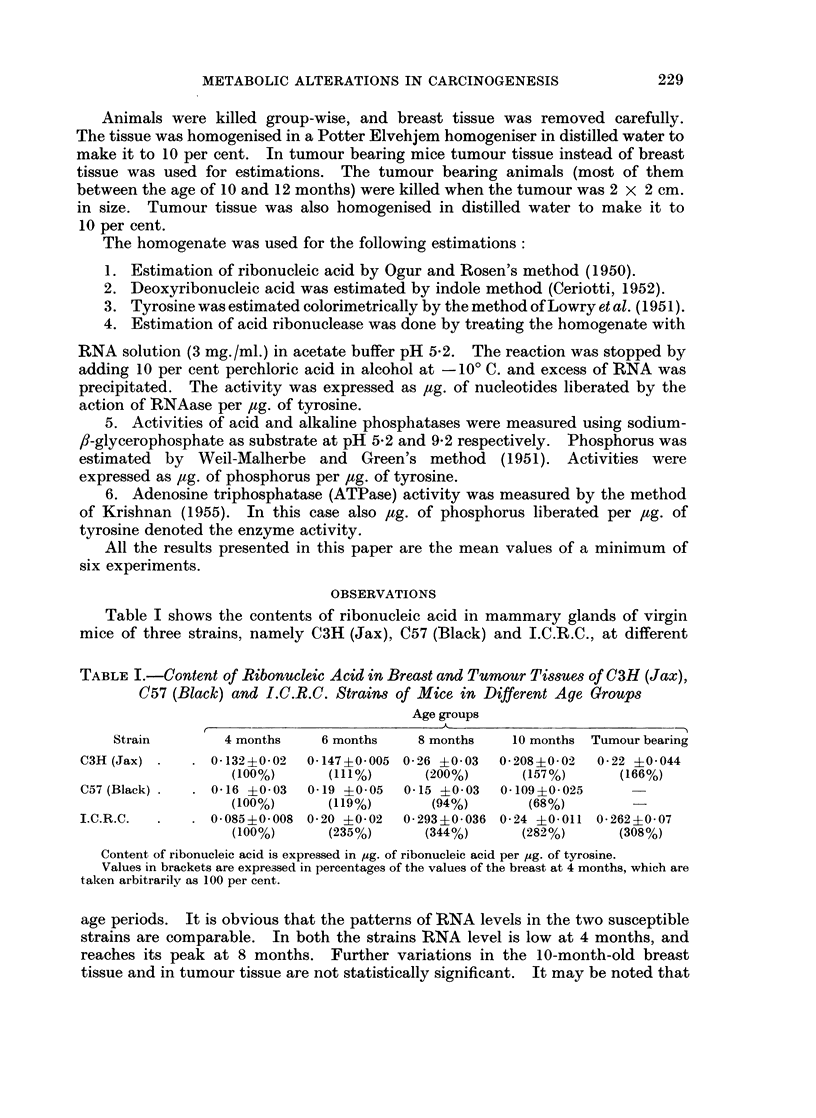

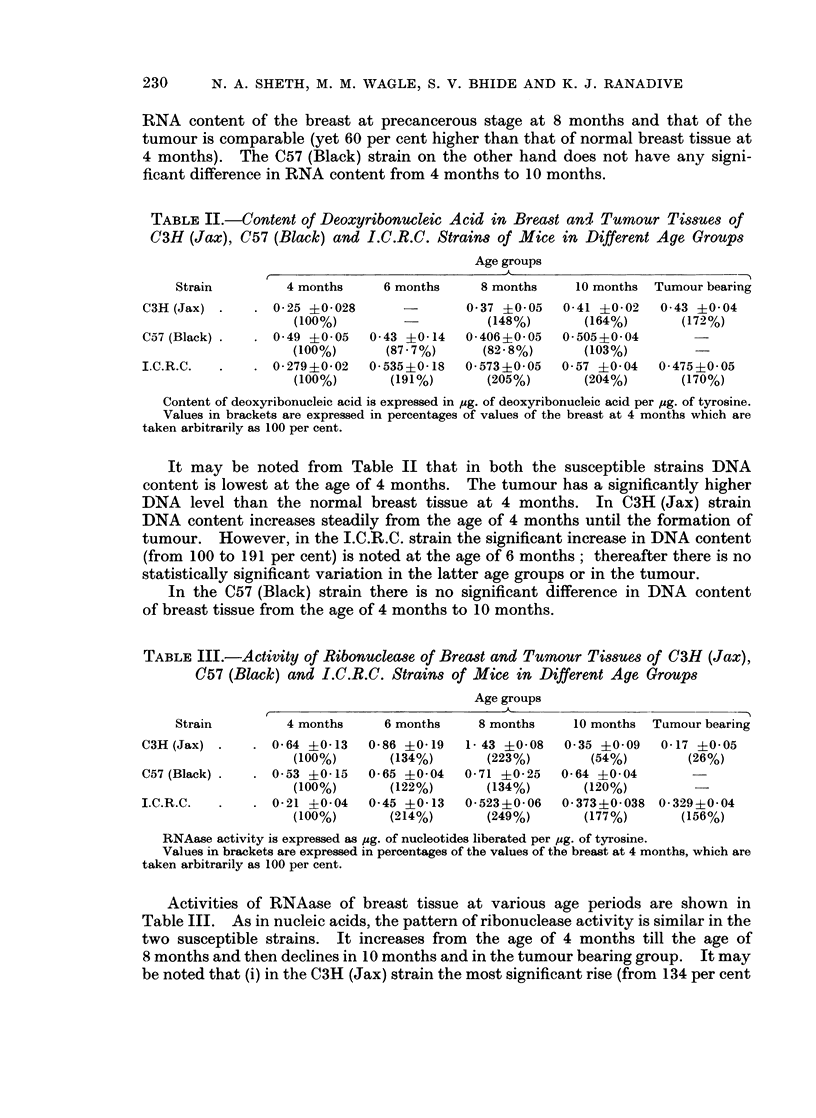

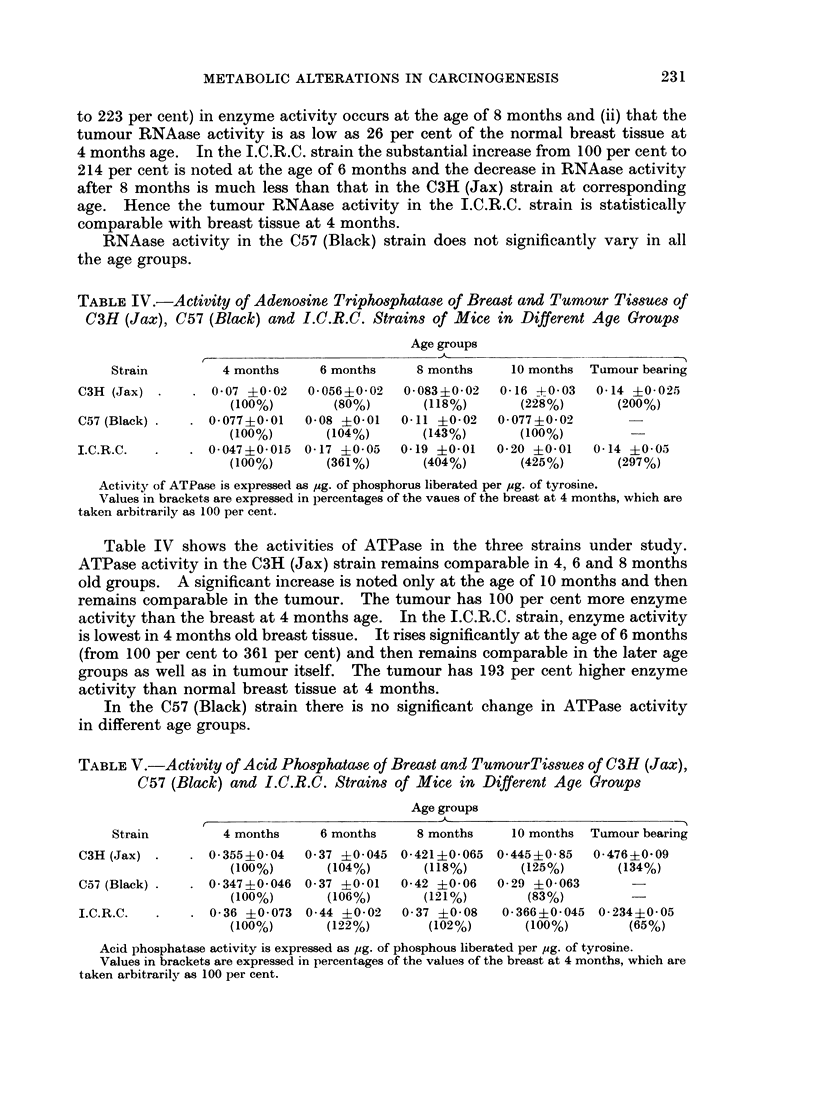

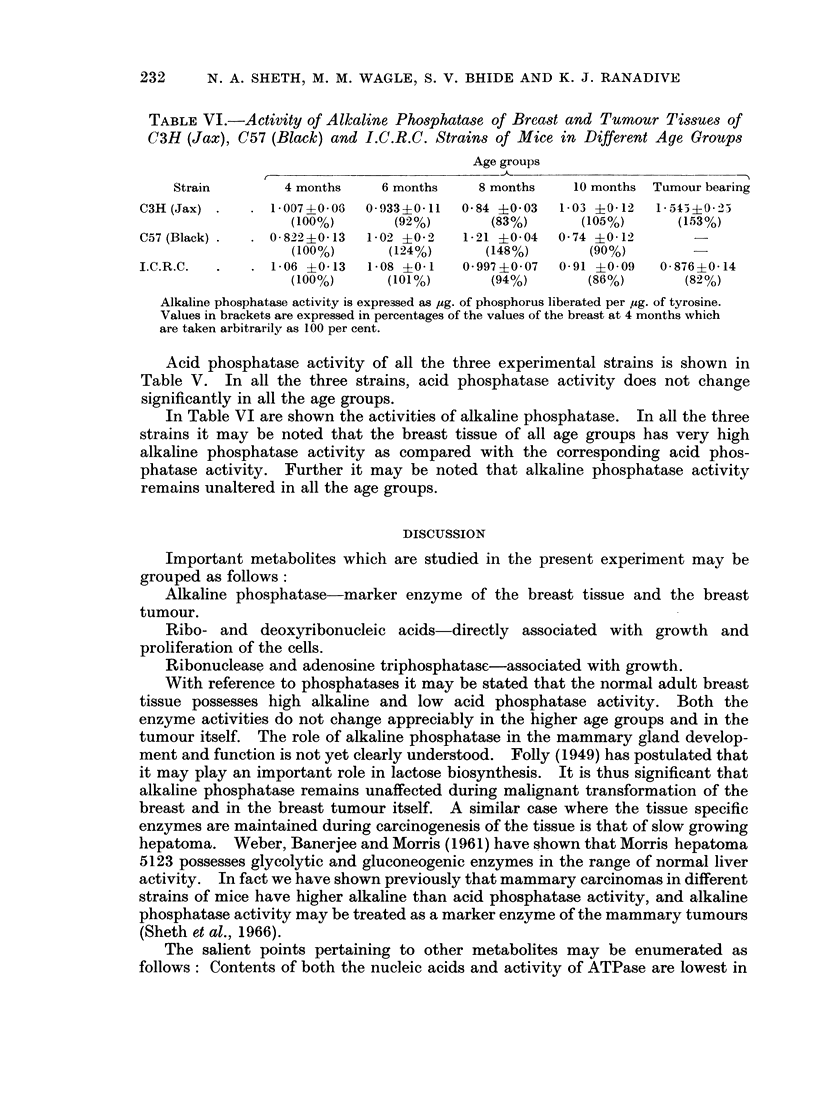

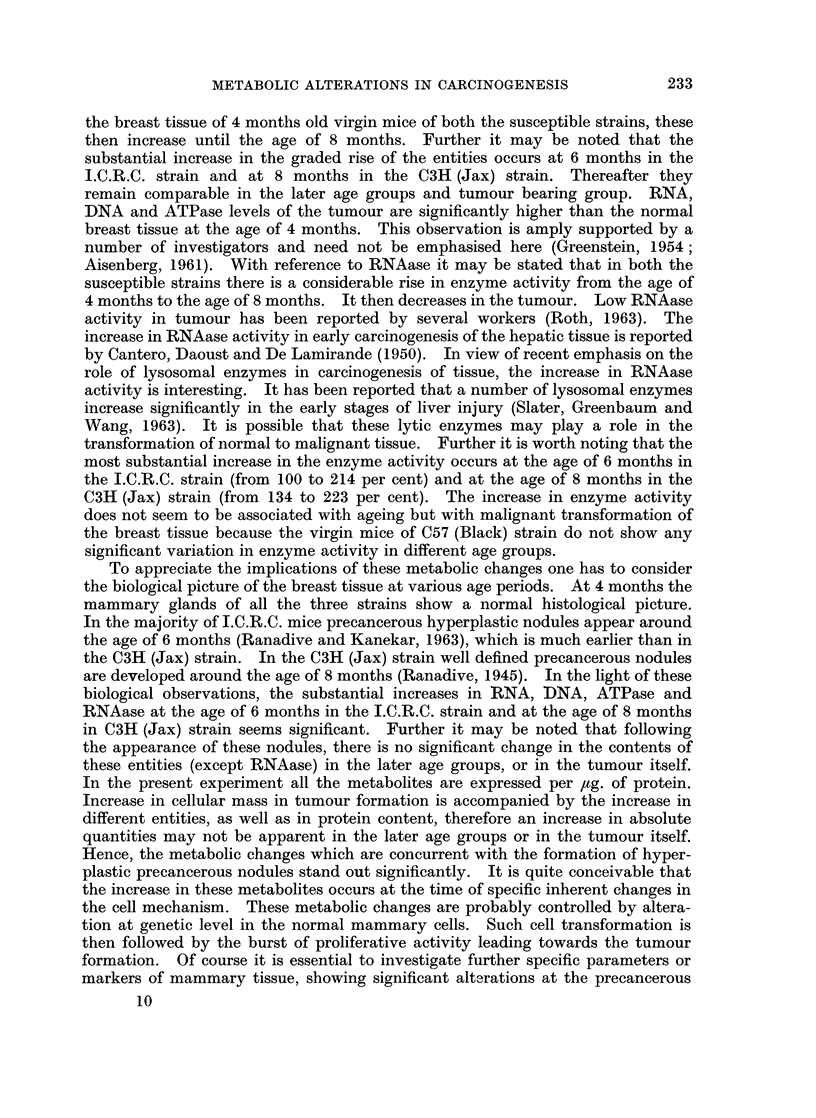

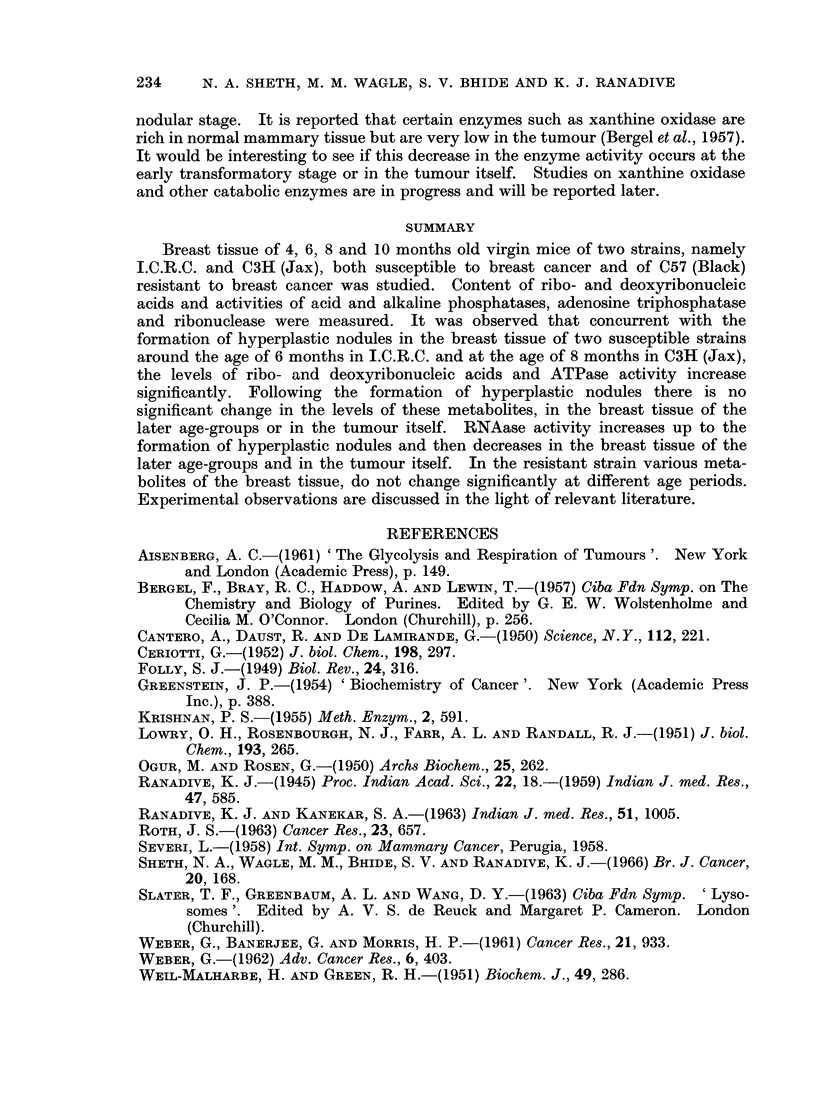


## References

[OCR_00422] CANTERO A., DAOUST R., de LAMIRANDE G. (1950). Nucleodepolymerase activity of precancerous rat liver.. Science.

[OCR_00424] CERIOTTI G. (1952). A microchemical determination of desoxyribonucleic acid.. J Biol Chem.

[OCR_00434] LOWRY O. H., ROSEBROUGH N. J., FARR A. L., RANDALL R. J. (1951). Protein measurement with the Folin phenol reagent.. J Biol Chem.

[OCR_00440] RANADIVE K. J. (1959). Biological studies on the mechanism of carcinogenesis.. Indian J Med Res.

[OCR_00443] RANADIVE K. J., KANEKAR S. A. (1963). BIOLOGICAL STUDIES ON THE NEW ALBINO MOUSE INBRED AT THE INDIAN CANCER RESEARCH CENTRE.. Indian J Med Res.

[OCR_00447] Sheth N. A., Wagle M., Bhide S. V., Ranadive K. J. (1966). An attempt at biochemical characterisation of histologically well defined tumours.. Br J Cancer.

[OCR_00456] WEBER G., BANERJEE G., MORRIS H. P. (1961). Comparative biochemistry of hepatomas. I. Carbohydrate enzymes in Morris hepatoma 5123.. Cancer Res.

[OCR_00457] WEBER G. (1961). Behavior of liver enzymes in hepatocarcinogenesis.. Adv Cancer Res.

[OCR_00459] WEIL-MALHERBE H., GREEN R. H. (1951). The catalytic effect of molybdate on the hydrolysis of organic phosphate bonds.. Biochem J.

